# Venous versus arterial iron administration in haemodialysis. Influence on erythrocytes antioxidant parameters

**Published:** 2015

**Authors:** CB Dogaru, C Capusa, L Gaman, E Torac, D Lixandru, M Gilca, L Iosif, C Muscurel, I Stoian, G Mircescu, V Atanasiu

**Affiliations:** * ”Carol Davila” University of Medicine and Pharmacy, Bucharest, Romania; ** R&D Irist Labmed, Bucharest, Romania

**Keywords:** haemodialysis, erythrocytes, antioxidant parameters

## Abstract

**Introduction** Intravenous iron administration in patients treated by haemodialysis for end stage renal disease can exacerbate oxidative stress by increasing the level of free redox active iron. A way to reduce the impact of iron on oxidative stress in haemodialysis patients may be the administration of iron through arterial extracorporeal circuit.

**Objective** The aim of our study was to compare the influence of iron route of administration (venous versus arterial extracorporeal circuit infusion) on antioxidant parameters in red blood cells of haemodialysis patients in order to clarify if arterial iron administration can have positive impacts related to iron induced oxidative stress.

**Method** Twenty stable patients on regular haemodialysis treatment were selected for the study. They were investigated in a cross-over design at 3 mid-week HD sessions, one week apart, without iron [HD basal] and with either IV infusion of 100mg iron sucrose over the first 20 minutes of HD session, via venous line [HDvenous], or the same solution infused on the arterial extracorporeal circulation [HDarterial].

Blood samples were drawn at 0 min, 40 min and 270 min. Erythrocytes superoxide dismutase (SOD), catalase (CAT), glutathione peroxidase (GSH-Px) activity, non-protein thiol levels and total antioxidant capacity (TEAC) were analysed.

**Conclusion** Haemodialysis significantly decreases the total antioxidant activity in erythrocytes. Iron supplementation, through venous or arterial extracorporeal route has no impact on the total antioxidant activity in red blood cells. Venous iron administration increases GPx activity in erythrocytes suggesting increased lipid peroxidation compared with arterial extracorporeal administration.

## Introduction

Haemodialysis associated oxidative stress has been well documented in scientific literature by now and is attributed mainly to dialysis membrane/ buffer bio incompatibility and activation of the immune response [**1**,**2**].

End stage renal disease is a condition frequently associated with iron deficient anaemia. Iron deficiency develops in chronic renal failure due to inadequate intake, deficient absorption, gastrointestinal bleeding, and blood loss within the dialyzer. Intravenous iron therapy is considered the most suitable method to treat anaemia in these patients.

Iron supplementation in patients treated by haemodialysis for end stage renal disease can exacerbate oxidative stress [**3**,**4**]. Pharmaceutical iron formulations used for parenteral administration usually contain a core of iron oxy-hydroxide gel surrounded by a complex carbohydrate shell. In plasma, the carbohydrate shell is destroyed by macrophages and the iron content is transported to the reticulo-endothelial system cells. Even rigorously checked pharmaceutical preparations may contain labile dialyzable iron [**5**]. Also, free, unbound iron may be released when the iron complex enters in contact with plasma, especially when transferrin is saturated [**6**]. Several studies have shown transferrin oversaturation after intravenous iron administration and a subsequent increase in unbound, redox active iron [**7**]. Uncomplexed or labile iron can generate toxic reactive oxygen species – Fenton and Haber Weiss reactions in specific conditions. The presence of increased oxidative stress was proved by increased superoxide generation [**8**], increased plasma total peroxide and malondialdehyde-MDA [**9**] and the induction of protein oxidation by intravenous iron administration [**10**].

Free redox active iron and increased oxidative stress may contribute to cardiovascular pathology in patients treated by haemodialysis [**11**].

Various attempts to reduce oxidative stress associated with iron administration in haemodialysis patients have been done. Different iron pharmaceutical preparations have been tested related to their ability to release free iron [**6**]. Promising results are shown by soluble ferric pyrophosphate [**12**]. Pre-treatment with antioxidants has been shown to reduce oxidative stress associated with iron administration in haemodialysis patients. The administration of 1200 IU of vitamin E, 6 hours prior the administration of 100 mg iron sucrose [**13**] reduced lipid peroxides. N-acetyl cysteine supplementation 10 days before iron administration also reduced lipid peroxidation [**14**]. Another way to reduce the impact of iron on oxidative stress may be the administration of iron through an arterial extracorporeal circuit. This extracorporeal arterial administration route may clear the labile iron from the pharmaceutical preparation in the first pass through the dialyser. The removal of large polysaccharides polymers is considered unlikely taking into account their high molecular mass [**15**], so the patient will receive the adequate dose of iron.

The aim of our study was to compare the influence of iron route of administration (venous versus arterial extracorporeal circuit infusion) on antioxidant parameters in red blood cells of haemodialysis patients in order to clarify if the arterial iron administration can have positive impacts related to iron induced oxidative stress.

## Subject and Methods

**Patient selection**

Twenty stable, non-smoker, hemodialysis (HD) patients on regular haemodialysis treatment for 73.38±68.57 months were selected for the study. Inclusion criteria were: without iron overload or inflammation, on steady dose of epoietin but no iron therapy at least one month before, ferritin level < 500 ng/ ml. They were investigated in a cross-over design at 3 mid-week HD sessions, one week apart, without iron [HD basal] and with either IV infusion of 100mg iron sucrose in 100ml 0.9% NaCl solution over the first 20 minutes of HD session, via venous line [HDvenous], or the same solution infused on the arterial extracorporeal circulation [HDarterial]. HD protocols consisted of three sessions per week, 4.5 h per session, with single-used low-flux polysulfone membrane dialyzers, bicarbonate dialysate and standard heparinisation.

The study was approved by the local medical Ethics Committee in compliance with the Declaration of Helsinki. 

Blood samples were drawn at 0 min, 40 min (20 minutes after the end of the iron infusion) and 270 min. Blood was sampled into litium-heparin vacutainer tubes and processed imediately after colection. Erytrocytes were separated by centrifugation at 1000G for 15 min, washed twice with 0,9% NaCl and hemolyzed with ice-cold deionized water. After centrifugation to remove red blood cells membranes, the supernatant was stored at -800C until analysis.

 Erythrocyte antioxidant defense was assesed spectrophotometrically by superoxide dismutase (SOD), catalase (CAT), glutathionperoxidase (GSH-Px) activity, non-protein thiol levels and total antioxidant capacity (TEAC).

Reagents and ultrapure water were treated with Chelex 100 (Merck, Darmstadt, Germany) to bind transitional metals. All reagents were of pure analytical quality and were purchased from Santa Cruz Biotechnology, Inc. (Dallas, Texas, USA). All assays were performed on a Perkin-Elmer Lambda EZ 210 (Perkin-Elmer, Boston, MA, USA) spectrophotometer.

**Measurement of erythrocyte antioxidant enzyme activities and total antioxidant capacity**

**Non-proteic thiols**

According to Beutler [**16**], erythrocyte non-protein thiols which mainly reduced glutathione (GSH), were measured. The erythrocyte lysate was mixed with a protein precipitationg reagent of metaphosphoric acid, disodium ethylenediamine-tetraacetic acid and sodium chloride dissolved in water. After 5 minutes, the mixture was filtered and 0.3M sodium phosphate and Ellman’s reagent were added. The absorbance was read at 412 nm against a reagent blank. The results were calculated by using the molar extinction coefficient for GSH of 13600 M-1cm-1 and were expressed as mmol/ g Hb.

**SOD activity**

SOD (EC. 1.15.1.1) activity was determined as described by Marklund [**17**], CuZnSOD from erythrocytes was extracted with an extraction reagent containing methanol: chloroform 62.7:37.5 (v/ v) stored at 2–8°C. After the addition of the extraction reagent, the mixture was briefly vortexed and centrifuged for 5 minutes at 3000 g and 4°C. The upper aqueous layer containing the enzyme was sampled. The rate of autoxidation of 2 mM pyrogallol in the reaction buffer (TRIS-cacodylic acid 50 mM, pH = 8.2, containing 1 mM DTPA) – with and without enzyme – was taken from the increase in absorbance at 420 nm. A unit of the enzyme is generally defined as the amount of enzyme that inhibits the reaction by 50%. Results are corrected for the dilution and expressed relative to Hb content.

**Erythrocyte CAT activity**

CAT (EC. 1.11.1.6) activity was measured by using the method described by Aebi [**18**]. The erythrocyte lysate was diluted in 0.05 M potassium phosphate buffer (pH=7), and the reaction was started by adding 10 mM hydrogen peroxide. Decrease in absorbance at 240 nm was measured for 30 seconds. Enzyme activity was calculated as a function of the rate constant of the first-order reaction (k), and was expressed as k per gram of Hb. 

**GSH-Px Activity**

The GSH-Px (EC 1.11.1.9) activity was indirectly measured by oxidation of NADPH to NADP+. 12 UI GSH reductase, 24 μM GSH and NADPH in 0,5 M TRIS-HCl buffer (pH 7.6) were added on the hemolysed diluted erytrocytes. The enzymatic reaction was initiated by adding cumene hydroperoxide as substrate. We followed the conversion of NADPH to NADP+ by a continous recording of the decrease in the absorbance at 340nm for 3 min. GSH-Px activity was expressed as U/ g Hb [**19**].

**Total antioxidant activity (TEAC)**

Total antioxidant activity was determined based on the 6-hydroxy-2,5,7,8 tetramethylchroman-2 carboxylic acid (Trolox, Sigma Aldrich Chemie, Munich, Germany) equivalent antioxidant capacity assay (TEAC) developed by Miller [**20**] with modifications [**21**]. The TEAC assay measures the relative abilities of antioxidants to scavenge the 2,2′-azino-bis (3-ethylbenzothiazoline-6-sulfonic acid) (ABTS) radical cation (ABTSx+), compared with the antioxidant potency of standard amounts of Trolox, the water-soluble vitamin E analogue. The ABTS radical was generated from the interaction between ABTS and potassium persulfate. Erythrocytes samples (10 μl) were mixed with 1 ml of 47 μM ABTSx+ and incubated for 1 minute at 30°C. Optical density (absorbance) was read at 734 nm against 5 mM phosphate buffered saline (pH 7.4). The percentage inhibition of absorbance, which is directly proportional to the antioxidant activity of the sample, was calculated. The assay was calibrated against a calibration curve with Trolox as standard. Plasma TEAC was expressed as millimoles per liter of Trolox, and RBC TEAC was expressed as micromoles of Trolox per gram Hb.

**Statistical analysis**

Data analysis was performed by using the GraphPad InStat software package (GraphPad Software, La Jolla, CA, USA). Differences between groups were computed by using analysis of variance parametric (Tukey’s) or nonparametric (Kruskal–Wallis) tests. A value of P < 0.05 was considered statistically significant.

## Results

**Fig. 1:**
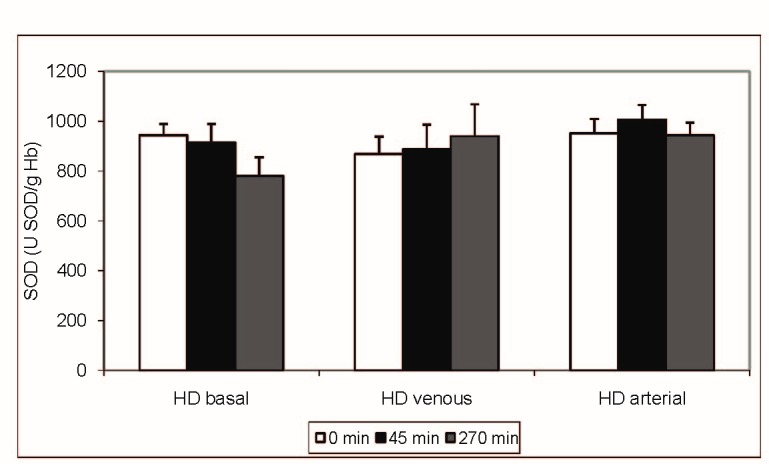
SOD (U SOD/ g Hb) - intradialytic variation of SOD levels

**Fig.2:**
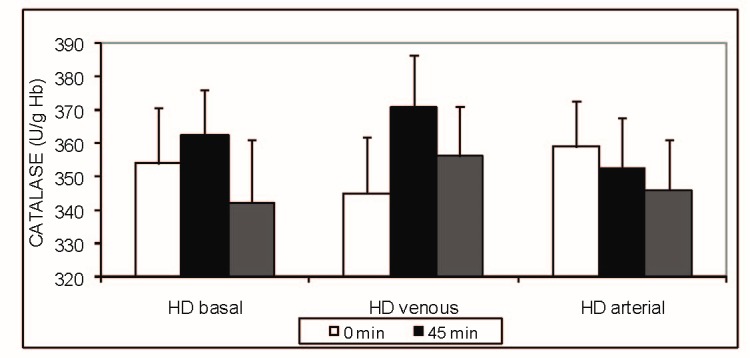
CATALASE (U/ g Hb) - intradialytic variation of CAT levels

**Fig. 3:**
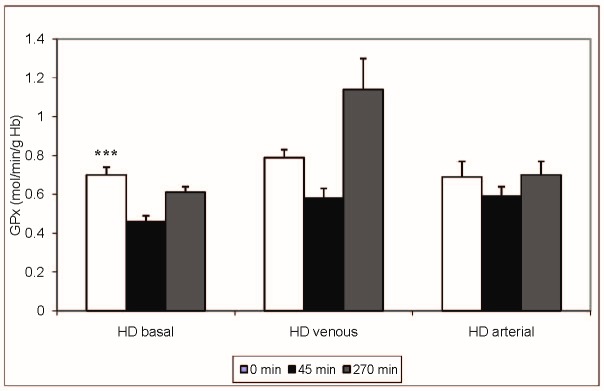
GPx (mol/min/g Hb) - intradialytic variation of GPx levels; [***0 min vs. 45 min – p < 0.001; **45 min vs. 270 min – p < 0.01; *45 min vs. 270 min – p < 0.05]

Results are expressed as mean + SD. No significant differences (p > 0.05, ANOVA) were found between the activities of the antioxidant enzymes SOD (**Fig. 1**) and CAT (**Fig. 2**) studied during the haemodialysis sessions. There was a significant difference in case of GPx activity (**Fig. 3**) at T3 (p < 0.01, ANOVA, Tukey-Kramer Multiple Comparisons Test), which was statistically higher for the venous iron administration (1,14 ± 0.75 mol/min/g Hb) when compared with basal (0.61 ± 0,16 mol/min/g Hb) or arterial administration (0,70 ± 0,31 mol/min/g Hb). Even if the GPx activity at T2 was higher for both the venous and arterial iron administration (venous 0.58 ±0.22, arterial 0.59 ± 0.22 versus 0.46 ± 0.17 mol/min/g Hb), when compared with the basal value, this difference did not reach the significance level (p = 0.0912, ANOVA). 

**Fig. 4:**
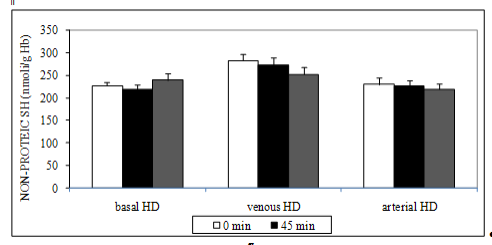
NON-PROTEIC SH (nmoli/ g Hb) – intradialytic variation of SH levels

**Fig. 5:**
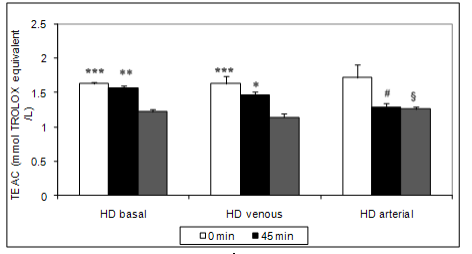
Intradialytic variation of TEAC levels; [***0 min vs. 270 min – p < 0.001; **45 min vs. 270 min – p < 0.01; *45 min vs. 270 min – p < 0.05, #0 min vs. 45 min – p < 0.05; §0 min vs. 270 min – p < 0.05]

Non-protein thiols (**Fig. 4**) were not significantly different (p > 0.05, t test) during the dialysis sessions. TEAC (**Fig. 5**) was statistically significantly lower at T2 (p < 0.05, basal 1,57 ± 0,16; arterial 1,46 ± 0,24; venous 1,29 ± 0,23 mmol Trolox/ L) and T3 (p < 0.05, basal 1,22 ± 0,21; arterial 1,14 ± 0,22; venous 1,26 ± 0,18 mmol Trolox/ L) versus T1 (basal 1,63 ± 0,12; arterial 1,14 ± 0,22; venous 1,72 ± 0,80 mmol Trolox/ L) for basal, arterial and venous iron administration. Even if the TEAC value at T3 was lower for the venous iron administration (1,14 ± 0,22 mmol Trolox/ L), there were no significant differences ( p > 0.05, ANOVA, Tukey-Kramer Multiple Comparisons Test) when compared with basal (1,22 ± 0.21 mmol Trolox/ L) or arterial (1,26 ± 0.18 mmol Trolox/ L) administration. 

## Discussion

Cells exposed to oxidative stress conditions usually have increased abilities to fight against free radicals. Erythrocytes are normally exposed to an increased concentration of free radicals due to haemoglobin autoxidation [**22**] As a consequence, red blood cells have a redutable antioxidant system which is able to protect them. Enzymatic antioxidants include superoxide dismutase SOD, catalase CAT, glutathione peroxidase GPx. Glutathione, GSH is one of the most important non enzymatic antioxidant present in red blood cells, being used as a cofactor for met-haemoglobin reductase, glutathione peroxidase and glutathione transferase. Oxidised glutathione is reduced by glutathione reductase, an enzyme by using NADPH as coenzyme.

Erythrocytes antioxidant systems also support the defence against free radicals generated inside the plasmatic compartment, the erythrocyte membrane being permeable for hydrogen peroxide, superoxide anion, nitric oxide and nitrates [**23**,**24**]. Plasma is relatively poor in enzymatic antioxidants so the red blood cell antioxidant systems also make them important in controlling the level of oxidative stress in blood.

Our study found statistical significant differences in SOD and CAT activities irrespective of the route of iron administration in haemodialysis treated patients. Also, no difference was noted in SOD and CAT activities for baseline HD session (without iron). Venous blood iron administration significantly increased glutathione peroxidase activity at 45 minutes and also at 270 minutes after the initiation of the haemodialysis procedure. This increased activity suggested an increased production of lipid peroxides by venous iron administration compared with arterial administration. Interestingly, the glutathione level was not significantly changed during the dialysis procedure, suggesting adaptive mechanisms able to compensate glutathione consumption by glutathione peroxidase.

Other studies found lower or higher SOD, GPx and glutathione after intravenous iron administration or after the haemodialysis procedure [**25**-**27**].

The conflicting results obtained could be explained by the different experimental designs: type of membrane, dialysate, type of administration procedure used (bolus versus infusion), type of pharmaceutical preparation.

Also differences between the initial ferritin levels among patients could differently influence erythrocytes enzyme activation after intravenous iron administration [**28**,**29**]. 

The total antioxidant capacity assays took into account the synergic actions of antioxidants and were considered more suitable than the individual antioxidant measurements in giving information related to the antioxidant reserve of biological systems.

In our study, erythrocytes antioxidant capacity has decreased significantly at the end of the dialysis sessions irrespective of the route of iron administration and also in basal conditions, without iron infusion. Increased oxidative stress associated with the haemodialysis procedure could explain this result. Taking into account that the antioxidant enzymes activities were not significantly changed for HD-basal and HD-arterial we could consider that small molecular weight antioxidants like vitamin E and C were mostly consumed to protect erythrocytes against oxidative stress in that situation.

As far as we know, there is no other study in literature that measures the total antioxidant activity in red blood cells, in the conditions of our study.

## Conclusion

Haemodialysis significantly decreases the total antioxidant activity in erythrocytes. Iron supplementation, through venous or arterial extracorporeal route has no impact on the total antioxidant activity in red blood cells. Venous iron administration increases GPx activity in erythrocytes, suggesting increased lipid peroxidation compared with arterial extracorporeal administration.

**Acknowledgement**

The work of Dogaru Beatrice Carmen was supported by the Sectorial Operational Programme Human Resources Development (SOP HRD) financed from the European Social Fund and by the Romanian Government under the contract number POSDRU/159/1.5/S/137390.
